# The use of metaverse in medical education: A systematic review

**DOI:** 10.1016/j.clinme.2025.100315

**Published:** 2025-04-15

**Authors:** Qian Li, Hui Duan, Xinxu Zhou, Xiaobin Sun, Lan Tao, Xiuying Lu

**Affiliations:** a The Third People's Hospital of Chengdu, Sichuan,610014, China; b University of Electronic Science and Technology of China, Sichuan,610054, China; c Department of operating theater, Sichuan Cancer Hospital & Institute, Sichuan Cancer Center, School of Medicine, University of Electronic Science and Technology of China, Chengdu, 610040, China

**Keywords:** Metaverse, Medical education, Systematic review, Virtual reality, Augmented reality, Mixed reality

## Abstract

**Objective:**

This study aims to systematically evaluate the influence of metaverse-based teaching tools on the medical teaching effect and the improvement of students' ability, providing evidence-based support for curriculum development.

**Methods:**

A comprehensive search of PubMed, Cochrane, Embase and Web of Science databases was conducted up to 9 March 2025. Two researchers independently screened articles, extracted data, and assessed bias using the Cochrane Handbook 5.1.0 checklist. Data analysis was performed using RevMan 5.3.

**Results:**

Thirty studies involving 1,560 participants. The metaverse was applied in various areas such as surgery training, anatomy teaching and communication skills. Common metaverse approaches include AR, VR, MR etc, with simulated teaching times ranging from as little as 10 min to lasting several weeks. Most studies reported improved student guidance, reduced training time, and enhanced learning interest, and confidence.

**Conclusion:**

Metaverse technology proves effective for medical teaching, offering theoretical support for further research.

## Highlights

• Based on the evidence, this study systematically searched and evaluated the randomised controlled studies on the application of meta-cosmic technology in medical teaching, and analysed its application effects, with a view to providing theoretical basis for clinical teaching, improving the availability and effectiveness of teaching, and reducing teaching costs.

## Graphical abstract

**Figure:**
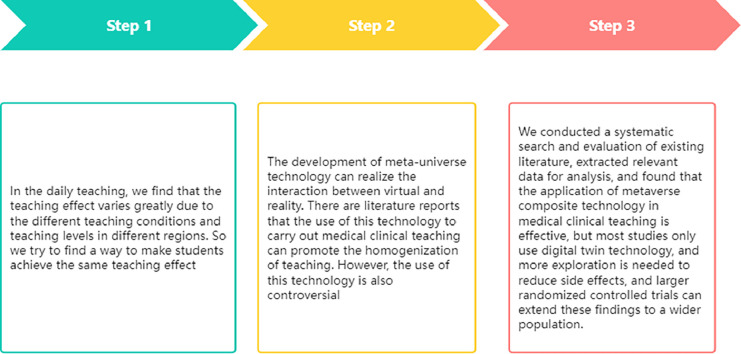

## Introduction

Due to constraints in working hours, regulations and ethical requirements, clinical teaching for medical students primarily relies on instructor demonstrations, theoretical explanations, and observation, which hinders the integration of theory with practice.^1^ In surgical education, allowing a novice surgeon to use a scalpel requires patience, knowledge, judgement and trust in the trainee’s ability.^2^ With the growing demand for healthcare and a persistent shortage of health professionals, alongside ethical considerations like patient safety and privacy, modern technology must be leveraged to diversify teaching methods and enhance effectiveness. Research has demonstrated that clinical simulations improve students’ technical and non-technical skills, learning motivation and the overall learning environment.^3^ The metaverse offers an immersive and innovative virtual world, utilising technologies like augmented, virtual or mixed reality to create a simulation experience that engages multiple senses.^4^

Originally proposed in the 1992 novel Snow Crash, the metaverse extends beyond a parallel dimension of the physical world, encompassing applications like gaming, social networking and training. It constructs a digital mirror of reality through code logic, forming a digital parallel universe that bridges virtual and physical realms. Leveraging advanced interactive technologies such as virtual reality (VR), augmented reality (AR) and mixed reality (MR), the metaverse offers users an immersive experience through devices like high-definition headsets and smart glasses. In this virtual realm, information is synchronised with reality, allowing user actions to create cross-domain influences and trigger new opportunities. While VR, AR and MR enhance perception and interaction in distinct ways, their boundaries are increasingly blurred. VR provides a fully virtual environment, AR overlays virtual information onto the real world, and MR integrates virtual and real elements, allowing interaction between both realms. Together, these technologies are driving the evolution of the metaverse.

The metaverse began to expand rapidly in 2021, penetrating various industries, including education^5^^,^^6^ and industrial design.^7^, ^8^, ^9^ In medicine, it shows significant potential, facilitating drug sensitivity testing and disease-specific health education,^10^ while revolutionising medical education through immersive, interactive virtual environments.^11^ For instance, the pulse pressure simulator in the metaverse precisely replicates the physiological characteristics of different age groups, enhancing palpation training.^12^ Its interactive nature transcends geographic and temporal boundaries, enabling real-time, synchronous learning experiences that greatly improve teaching outcomes.^13^ The study demonstrated that metaverse-based career guidance significantly boosts nursing students’ confidence in career decision-making, affirming its effectiveness in professional training.^14^ From neurosurgery to ophthalmology, radiology to orthopaedics, and even nursing skill development, the metaverse is enriching medical education like never before. With high-fidelity graphics and visualisation technologies, learners engage in hands-on practice, advancing medical education to new heights.^15^, ^16^, ^17^, ^18^, ^19^, ^20^, ^21^, ^22^, ^23^, ^24^, ^25^, ^26^, ^27^, ^28^, ^29^, ^30^

Despite its growing use in medical education, metaverse applications vary in interventions, target populations and outcomes. Currently, no comprehensive review offers a holistic view of its educational applications. Thus, this systematic evaluation reviews existing randomised controlled studies on metaverse applications in medical education. It summarises the current status, benefits and limitations of these applications, offering insights for future educational strategies in medical education.

## Materials and methods

### Search strategy

This systematic review was registered in PROSPERO (https://www.crd.york.ac.uk/PROSPERO/), the international database for registered systematic reviews, under the registration number CRD42023446864.

A systematic literature search was conducted up to 9 March 2025, utilising databases such as PubMed, Cochrane CENTRAL, Embase and Web of Science. The MeSH terms were “Metaverse” “Education, Medical”, “Augmented Reality” and “Virtual Reality”, and are searched in combination with other free words such as Mixed Reality, AR, VR, Medical Education. A comprehensive literature search was conducted in the PubMed database as following: (((Metaverse) OR (((“Augmented Reality”[Mesh]) OR ((((((((Augmented Realities) OR (Realities, Augmented)) OR (Reality, Augmented)) OR (Mixed Reality)) OR (Mixed Realities)) OR (Realities, Mixed)) OR (Reality, Mixed)) OR (artificial reality))) AND (((((((((((((Virtual Reality[MeSH Terms]) OR (Reality, Virtual)) OR (Virtual Reality, Educational)) OR (Educational Virtual Realities)) OR (Educational Virtual Reality)) OR (Reality, Educational Virtual)) OR (Virtual Realities, Educational)) OR (Virtual Reality, Instructional)) OR (Instructional Virtual Realities)) OR (Instructional Virtual Reality)) OR (Realities, Instructional Virtual)) OR (Reality, Instructional Virtual)) OR (Virtual Realities, Instructional)))) AND (((((Education, Medical[MeSH Terms]) OR (Medical Education)) OR (Teaching)) OR (Training)) OR (Learning))) AND ((((((cross-sectional) OR (cohort)) OR (randomised controlled trial)) OR (RCT)) OR (trial)) OR (random*)), and the search strategy of other databases was detailed in Supplementary information. Subsequently, a manual search was conducted to incorporate references that were acknowledged as Table S1 in the literature.

### Inclusion exclusion criteria

Studies were selected based on defined inclusion and exclusion criteria.

Inclusion criteria: (1) Study type: randomised controlled trials (RCTs) related to medical pedagogy; (2) Study demographic: Medical personnel or students; (3) Instructional technology: Use of metaverse elements, including augmented reality (AR), virtual reality (VR) and mixed reality (MR); (4) Outcome metrics: Studies reporting relevant outcomes.

Exclusion criteria: (1) Repetitive or redundant publications; (2) Non-English literature; (3) Non-primary research, including conference summaries, letters, editorials and responses.

### Literature screening and data extraction

Using Endnote, duplicates were removed from the literature. Two researchers, Q.L and X.Z, independently evaluated the remaining studies based on predetermined inclusion and exclusion criteria. A preliminary review of titles and abstracts was followed by a thorough full-text assessment to exclude ineligible articles. In case of disagreement, a third researcher, H.D, was consulted to reach a consensus.

Data extraction from the selected studies was conducted systematically, covering: (1) Authors; (2) study period; (3) Country; (4) Teaching scene; (5) Teaching object; (6) Participants; (7) Intervention; (8) Outcome.

### Quality assessment

The quality of the randomised controlled trial (RCT) literature was assessed based on the Cochrane Handbook for Systematic Reviews of Interventions 5.1.0. The evaluation included seven domains: random sequence generation, allocation concealment, participant and personnel blinding, outcome assessment blinding, completeness of outcome data, selective reporting, and other potential biases.^31^ Each domain was rated as low, high or unclear risk. Studies with more ‘low-risk’ ratings were considered high quality. Two researchers (Q.L and X.Z) independently conducted the assessments, and disagreements were resolved by a third researcher (X.L).

## Results

### The literature searching and basic information of included articles

**Fig. 1:**
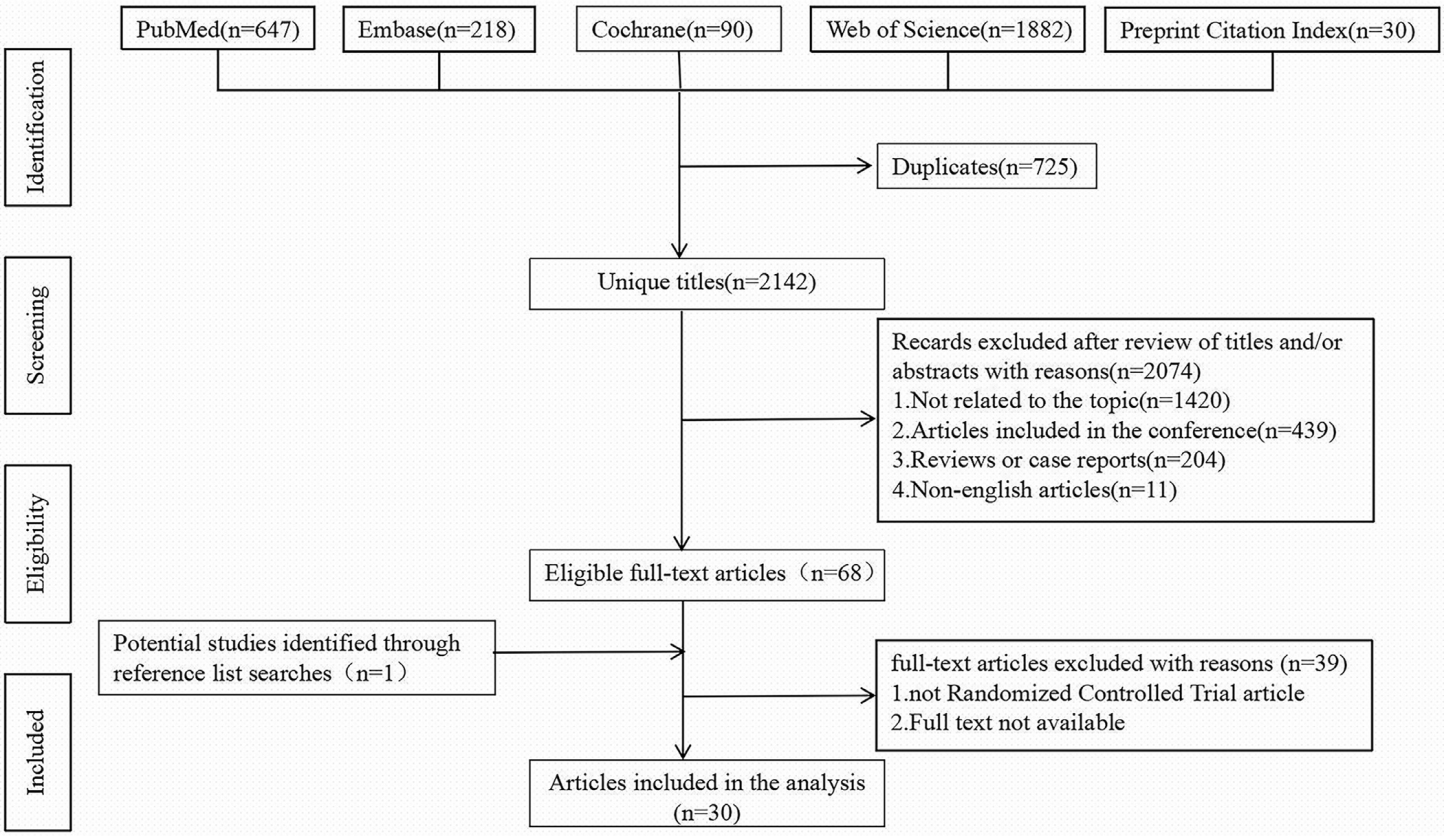
Flow chart of article systematic retrieval and selection.

Our study focused on the use of augmented reality (AR), virtual reality (VR), and mixed reality (MR) in medical education via randomised controlled trials (RCTs), excluding reviews, surveys and case–control studies. A total of 30 articles were included (Fig. 1), spanning from 2012 to 2025, with most originating from the USA, South Korea and Europe. Among these, 23 studies involved medical students, four involved doctors, one involved nurses, and two included both medical staff and students. The applications covered surgical training (n=11), ophthalmic surgery (n=1), theoretical knowledge and nursing practice (n=12), anatomy teaching (n=4), radiology teaching (N=1) and communication training (n=1). One study used a metaverse virtual simulation for teaching, eight used MR, four used AR, and 15 used VR. Table 1 provides the list of included articles.

**Table 1:** Characteristics of include studies.

Authors	Study period	Country	Teaching scene	Teaching object	Participants (intervention group / control group)	Intervention	Outcome
Greuter *et al*^16^	2018–2020	Switzerland	Cerebrovascular anatomy	Neurosurgery residents and medical students (4th–6th years)	40/40	VR	Aneurysm detection time; Accuracy of describing the anatomical and spatial features of the aneurysm
Bogomolova *et al*^17^	2018	The Netherlands	Anatomy	First- and second-year undergraduate students of medicine and biomedical sciences	20/(20/18)	AR	Visual-spatial abilities; Anatomy knowledge; Evaluation of learning experience
Stojanovska *et al*^18^	2017	USA	Musculoskeletal anatomy	Second-year medical students	31/33	MR	Curricular time; Exam score
Liu *et al*^19^	2020	China	Understanding of the pulmonary lesions caused by COVID-19	Radiologists, surgeons and medical students	30/30	MR	Task scores; NASA Task Load Index Scores; Likert-scale questionnaire scores
Rai *et al*^20^	2012	Canadian	Binocular indirect ophthalmoscopy	Postgraduate year 1 (PGY1) ophthalmology residents	13/15	AR	Time required; Total score; Performance
Logishetty *et al*^21^	2019	UK	Total hip arthroplasty (THA) surgical training	Surgical trainees (postgraduate years 3–5)	12/12	VR	Procedure-based assessment (PBA) global summary scores; The number of satisfactory components in the expert-assessed PBA; Technical performance and operative time
Blumstein *et al*^22^	2020	USA	Tibial intramedullary nailing (IMN) procedure	First- and second-year medical students without prior experience of procedure	10/10	VR	Aggregate global assessment scores; Percentage of steps completed correctly; Completion of later steps; Scored in knowledge of instruments; Average improvement
William *et al*^23^	2022	USA	Reverse total shoulder arthroplasty	Junior orthopaedic surgery residents (postgraduate year (PGY) 1–3)	6/8	VR	Objective structured assessment of technicals kill (OSATS); Global rating scale; Time to completion of assessment; Post-training written knowledge score
Lohre *et al*^24^	2020	Canada	Orthopaedic education	Orthopaedic surgical residents and orthopaedic shoulder arthroplasty surgeons	12/11	VR	OSATS score; Knowledge score; Time to completion of the cadaveric task.
McKinney *et al*^25^	2019–2020	USA	Unicompartmental knee arthroplasty (UKA)	Orthopaedic surgery residents	11/11	VR	Programme operation score; Time spent in study and procedural Participant Questionnaire
Orland *et al*.^26^	2020	USA	Tibial intramedullary nailing (IMN) procedure	First- and second-year medical students	(8/9)/8	VR	Proportion of participants who completed the task; Proportion of incorrect steps; The number of hints requested during the test; Mean time to completion of the task
Birrenbach *et al*^27^	2020	Switzerland	COVID-19-related skill	Medical students	15/14	VR	Number of missing areas during hand disinfection; Nasopharyngeal swab test score; Number of contaminated body areas during doffing; Usability, satisfaction, simulator sickness, sense of presence, and immersion
Heo *et al*^28^	2022	Korea	Mechanical ventilator setup	Nurse	15/15	AR	Overall score of the procedure; The level of assistance required; Confidence, suitability, and whether they intended to recommend AR system to others
Plotzky *et al*^29^	2023	Germany	Endotracheal suctioning skill	Undergraduate nursing students	47/(43/41)	VR And MR	Knowledge test; Objective structured clinical examination (OSCE); Satisfaction with learning; Acceptance of VR technology; Qualitative data
Schoeb *et al*^30^	2017–2018	Germany	Male catheterisation	The students of 4th and 5th year in a 6-year MD (medical doctor)	59/105	MR	Teaching preference and learning experience; Self-evaluation; Objective structured clinical examination (OSCE); Standardised system usability scale
Tadlock *et al*^32^	2020	USA	Combat casualty care-related procedures	Special forces medics, non-surgeon physicians, and junior surgeons	8/6	MR	Likert scale-based questionnaire
Wang *et al*^33^	2020	New Zealand	Anatomy	Second-year medical students	19/(15/18)	MR	Minute-by-minute EEG signals; Anatomy test; User perception and usability survey; Memorability and long-term retention survey; psychometric tests
Nagayo *et al*^34^	2021	Japan	Suture techniques in open surgery	Medical students	19/19	AR	Global rating (GR) form; Task-specific (TS) checklist; System usability measure; Level of confidence and interest
Pulijala *et al*^35^	2017	UK	Le Fort I osteotomy	Novice surgical residents	51/40	VR	Evaluation scores of the perceived self-confidence levels; Changes in the knowledge levels; Perceived self-confidence scores
Veer *et al*^36^	2022	Australia	Asthma medical knowledge	First-year undergraduate students	33/34	MR	Knowledge retention scores after 2 weeks; Participant perceptions
Liaw *et al*^42^	2018	Singapore	Nurse–physician communication team training	Third or fourth year of medicine or nursing students	60/60	VR	Team communication performance; Teamwork attitudes
Yang *et al*^43^	2022	South Korea	Early-onset schizophrenia nursing programme	Third-year nursing students	29/29	Metaverse	Knowledge,; Critical thinking ability; Learning self-efficacy; Communication ability; Learning satisfaction and confidence
Francis *et al*^46^	2020	USA	Operating room simulation teaching	Second year physician assistant students	26/26	VR	Measuring self-efficacy
Lamb^44^	2023	USA	Tibia intramedullary nail (IMN) procedure	Fourth-year medical students	19/19	VR	Global assessment five-point scale assesses overall performance; Evaluate satisfaction, confidence, applicability and overall interest
Alam^37^	2024	India	Lumbar pedicle screw insertion (LPSI)	Residents and fellows	19/19	VR	The accuracy of the placement of lumbar pedicle screws
Guha^45^	2023	UK	Basic arteriotomy and closure	Clinical medical student	18/18	MR	Overall proficiency score; Score for instrument selection; Suture quality; Participant learning style and performance; Participant feedback
Çetinkaya Uslusoy^38^	2023	Turkey	Course ‘Nursing Process’	First-year students studying in the Department of Nursing	20/22	Metaverse	Instructional materials motivation survey (IMMS); Academic success assessment (quiz)
Brix^39^	2024	Denmark	Basic life support	Third-year nursing students	30/29	VR	Self-efficacy and professional competence; Interviews
Dubinski^40^	2025	Rostock	Familiar with operating room techniques and environment	Medical students	12/12	VR	The number taking the wrong direction; Dress in surgical gown; The number getting stuck
d’Aiello^41^	2022	Italy	Congenital heart disease	Medical students	19/(20/20)	MR	Proficiency survey; Satisfaction regarding

### The result of quality assessment

**Fig. 2:**
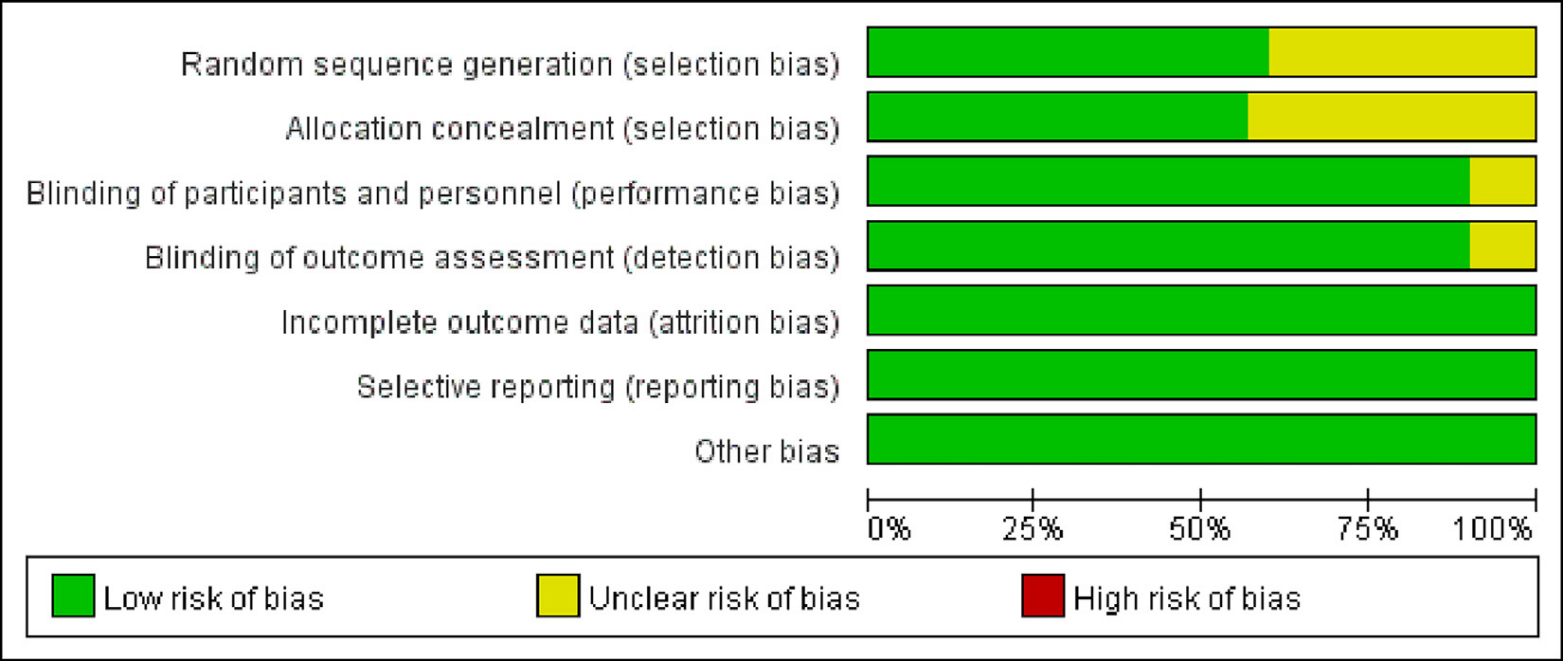
Risk of bias graph.

**Fig. 3:**
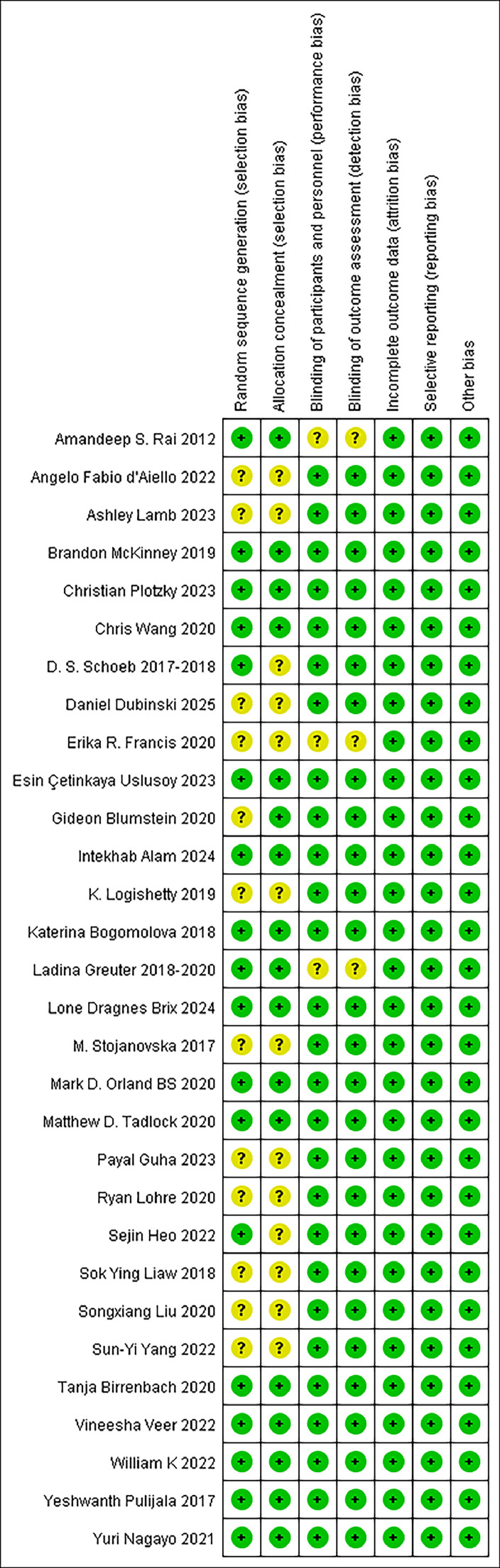
Risk of bias summary.

A quality assessment revealed that 18 studies generated randomised sequences,^16^^,^^17^^,^^20^^,^^23^^,^^25^, ^26^, ^27^, ^28^, ^29^, ^30^^,^^32^, ^33^, ^34^, ^35^, ^36^, ^37^, ^38^, ^39^ and 17 studies implemented allocation concealment.^16^^,^^17^^,^^20^^,^^22^^,^^23^^,^^25^, ^26^, ^27^^,^^29^^,^^32^, ^33^, ^34^, ^35^, ^36^, ^37^, ^38^, ^39^ Eight studies lacked blinding, but outcome assessments were unaffected.^17^^,^^28^^,^^33^^,^^37^, ^38^, ^39^, ^40^, ^41^ Blinding of patients and principal investigators was maintained in three studies, with no risk of unblinding.^18^^,^^24^^,^^25^ Blinding of outcome measures was used in 16 studies.^19^^,^^21^, ^22^, ^23^^,^^26^^,^^27^^,^^29^^,^^30^^,^^32^^,^^34^, ^35^, ^36^^,^^42^, ^43^, ^44^, ^45^ Four studies provided details on sample loss and reasons for attrition, with outcome data generally complete.^17^^,^^22^^,^^24^^,^^35^ The remaining studies reported complete data, with primary and secondary outcomes thoroughly described, and no other bias was detected. The quality assessment results are presented in Fig. 2, Fig. 3.

### Meta-universe in teaching medical students

Meta-universe technology is increasingly influencing medical education, offering an immersive learning environment for students. Yang *et al*^43^ employed this technology for immersive simulation in nursing care of early-onset schizophrenia, reporting significant improvements in students’ knowledge, critical thinking and communication skills compared to traditional online lectures. Similarly, Stojanovska *et al*^18^ used Microsoft HoloLens for MR visualisation in anatomy teaching, cutting teaching time nearly in half while seamlessly integrating art with education. Bogomolova *et al*^17^ demonstrated that HoloLens augmented reality is particularly effective for students with poor mental rotation skills, making the learning process more appealing. Blumstein^22^ focused on orthopaedic surgery training for students with no surgical experience, showing that VR technology led to substantial improvements in intramedullary nail surgery technique compared to traditional printed guide-based teaching. Overall, meta-universe teaching enhances the learning experience, accelerates knowledge acquisition and improves practical skill development.

### Meta-universe in the teaching of medical personnel

As an innovative tool for medical education, the connectivity and interactivity of the metaverse have significantly optimised medical training and enriched teaching practices. Pulijala *et al*^35^ applied VR in maxillofacial surgical skills training for novice hand surgery residents, enabling them to practise complex procedures in a risk-free virtual environment. This approach notably improved both their confidence and skills compared to traditional two-dimensional methods. Similarly, Tadlock *et al*^32^ introduced mixed reality (MR) technology in the training of special forces non-surgeons, utilising HoloLens for real-time holographic guidance from experts. This enhanced teacher–student interaction and feedback, suggesting potential for future telemedicine education. Heo *et al*^28^ demonstrated the efficacy of AR in respiratory training for nurses, showing improvements in learning efficiency, reduced help-seeking and increased confidence. Collectively, metaverse-based teaching not only reinforces medical fundamentals but also offers new avenues for skill enhancement and confidence-building among medical professionals.

### Application of meta-universe in different teaching scenarios

#### Meta-universe-based simulation teaching

Innovative metaverse models in medical teaching accelerate skill acquisition among students and foster teamwork. Rai *et al*^20^ introduced EyeSI AR simulators in ophthalmology residency training, demonstrating their superiority over traditional methods in enhancing resident skills. Liaw *et al*^42^ found that VR environments effectively improved communication and collaboration in medical student teams. Francis *et al*^46^ showed that VR in operating room simulations significantly increased students’ self-efficacy. Collectively, these studies highlight the metaverse’s vast potential for improving diverse skills in complex medical simulations.

#### Metaverse opens new path for surgical training

The meta-universe technology holds significant promise in medical education, particularly in orthopaedic surgical training. Virtual reality (VR) not only accelerates learning and skill refinement, but also enhances the overall learning experience. Logishetty *et al*^21^ demonstrated that VR training for total hip arthroplasty (THA) led to superior outcomes compared to traditional methods, including better overall scores, reduced operative time and improved accuracy. Similarly, McKinney *et al*^25^ found that VR training for unicompartmental knee arthroplasty (UKA) resulted in higher accuracy, faster completion, and an improved learning experience compared to conventional training. Orland *et al*^26^ reported that VR was more effective than traditional graphic materials for teaching tibial intramedullary nail insertion, enhancing both efficiency and accuracy. Lohre *et al*^24^ showed that VR outperformed journal reading for learning glenoid exposure, providing greater realism and improved manoueving skills. Although William *et al*^23^ did not find a significant difference in OSATS scores between VR and traditional methods for reverse-T shoulder arthroplasty, the immersive nature of VR still offers valuable advancements in surgical training.

#### Metaverse applied to teaching radiological knowledge

In orthopaedic surgery education, virtual reality (VR) is widely utilised, while mixed reality (MR) is increasingly applied in radiology instruction. Integrating meta-universe technology into medical teaching enhances student engagement through immersive and interactive learning experiences, potentially optimising educational outcomes. Veer *et al* explored MR technology in teaching asthma medical knowledge by developing a Unity 3D platform that enables students to interact with 3D models of the lungs and heart using gestures and voice commands. This MR-based approach received higher ratings for experiential quality, learning efficiency and recommendation willingness compared to traditional graphic materials.^36^ MR not only maintains effective knowledge transfer but also enhances learning appeal and interactivity, offering practical insights for making medical teaching more efficient, intuitive and engaging.

#### Metaverse applied to knowledge and skill enhancement training

In medical education, meta-universe technology enhances students’ professional knowledge and skills. Schoeb *et al* applied MR technology in bladder catheterisation training, significantly improving students’ operative skills, providing a reference for future studies.^30^ Birrenbach *et al*^27^ used VR to optimise COVID-19 nasopharyngeal swab collection training, showing that the experimental group outperformed the control in performance scores (scoring a median of 14 vs 12), with higher ratings for immersion, tolerance and satisfaction. These studies demonstrate the effectiveness of immersive technologies in improving procedural skills. Liu *et al*^19^ found that MR-based teaching significantly improved students’ cognitive ability to recognise COVID-19 lung lesions. Nagayo *et al*^34^ explored AR technology for suturing technique training and found that the motion provided in the AR system was more helpful for manipulating surgical instruments compared to the video. Plotzky *et al*^29^ reported that while VR did not significantly improve endotracheal suctioning success rates compared to the control group, the VR group showed higher levels of cognitive satisfaction, ease of use and enjoyment (15.72 vs (19.93 and 17.96)). The application of meta-universe technologies across basic and advanced skill training enhances hands-on skills, expertise and learning satisfaction, despite accompanying technical and practical challenges.

#### Applying the metaverse in teaching anatomy

Anatomy is a crucial area in medical education. Wang *et al*^33^ applied MR technology to a neuroanatomy course, showing that while text-based instruction outperformed 3DM in the short term, knowledge retention was more sustained over time in students taught with MR. Greuter *et al*^16^ used VR to teach neurosurgical cerebrovascular anatomy, and the study demonstrated that the immersive environment of VR significantly reduced the time to aneurysm detection, with most participants favouring VR for anatomical knowledge acquisition. While these studies emphasise the advantages of MR and VR in improving learning outcomes, they also indicate potential drawbacks such as dizziness or nausea during use.

## Discussion

The rapid advancement of technology has led to the widespread influence of the metaverse on human life and work patterns, garnering significant attention in medical education research.^47^ Compared to traditional models – such as laboratory practice, clinical internships and classroom teaching – the metaverse offers a novel approach to medical education with its benefits of resourcefulness, cost-effectiveness, safety, convenience and efficiency.^5^^,^^18^^,^^48^ In the metaverse’s virtual environment, students can perform virtual surgeries, engage in simulated case discussions, and practise communication skills without impacting real patients, thereby enhancing practical skills and teamwork abilities.^42^^,^^43^^,^^49^ Additionally, metaverse-based teaching enables the collection and analysis of students’ learning behaviour data, allowing educators to tailor personalised learning resources and course content, thus improving educational management and personalising the learning experience.^13^

Research on the application of metaverse technology in medical education is extensive; however, the interventions, research targets and conclusions across studies lack a unified standard. This study synthesises and systematically evaluates the use of the metaverse in medical teaching, finding a consensus that supports its positive potential in this field.^29^ Compared to traditional teaching methods, metaverse-based instruction enhances teaching effectiveness, significantly shortens the teaching cycle,^16^^,^^18^ and transcends temporal and spatial constraints.^43^ Its unique three-dimensional visual environment offers learners unprecedented immersion to explore complex medical knowledge and skills.^16^^,^^17^^,^^19^^,^^34^^,^^50^ Such immersive experiences effectively boost students’ interest in learning and enhance their self-confidence and self-efficacy.^28^^,^^29^^,^^35^^,^^46^ Moreover, the metaverse provides more standardised instruction^22^ and improves the retention and application of knowledge among medical students.^34^

In anatomy education, although cadaveric dissection offers unmatched authenticity and intuition, its limitations include resource scarcity and high costs. The meta-universe introduces a virtual platform that allows students to practise before engaging with physical specimens. This approach not only reduces costs but also enables unlimited repetition of knowledge, enhances understanding of anatomical space, and achieves teaching outcomes comparable to or even superior to cadaveric dissection.^15^^,^^16^^,^^18^

In surgical education, the metaverse demonstrates significant potential. It offers a platform for practising rare and complex surgeries as well as for novice surgeons, providing a virtual environment that simulates both complications and routine cases. This set-up enables repeated practice under safe conditions, enhancing emergency response, surgical precision and operational efficiency.^21^^,^^25^^,^^35^ Additionally, the metaverse allows for precise capture of fine motor movements, such as finger positioning, facilitating real-time monitoring and guidance by educators.^32^^,^^51^ This capability improves surgical accuracy^23^ and enhances students’ learning experience and satisfaction. Furthermore, studies by Lorenzo-Alvarez^52^ show that the metaverse’s gamified learning environment, with realistic graphics and visual interactions, overcomes the limitations of traditional 2D materials and greatly boosts students’ learning enjoyment and knowledge retention.

The metaverse, functioning as a ‘simulated workplace’, allows students to familiarise themselves with the future healthcare environment through near-realistic simulations of consultations, medical communication and hands-on practice, facilitating a smooth career transition.^53^^,^^54^ In first aid training, metaverse-based scenarios offer realistic simulations, enabling learners to train efficiently anytime and anywhere while reducing teaching costs.^55^ Additionally, the metaverse supports patient education and health management through digital twins, providing similar high-quality training scenarios.^56^ In English teaching, the metaverse creates virtual environments that offer immersive and interactive learning experiences for both teachers and students, meeting their educational needs in both real and virtual settings, and enhancing student interactivity, immersion and cognitive engagement.^6^

The integration of the metaverse into medical education is becoming increasingly prevalent, utilising VR/AR/MR technologies to enhance teaching and learning. This method transcends physical limits, providing an immersive educational experience. However, the metaverse, especially through digital twin technology, fails to fully replicate real-world interactions, such as nuanced facial expressions.^43^ Additionally, users may experience discomfort, including dizziness and nausea, while engaging with the metaverse.^16^ These challenges underscore the complex, interdisciplinary nature of metaverse development, requiring continual technological advancements. Moreover, the metaverse raises significant concerns regarding mental health, privacy and adherence to medical ethics.^57^ Addressing these multifaceted issues, particularly in technological refinement and ethical considerations, is essential for the effective integration of the metaverse into medical education.

This study has several limitations. The variability of indicators across studies results in a lack of uniformity, making meta-analysis unfeasible. Moreover, the small sample sizes in the included studies may introduce selection bias, and the impact of the metaverse on medical education with larger, more representative samples remains unexplored. Thus, the applicability of the metaverse in medical education, particularly with larger populations, requires further investigation.

Most research on the metaverse’s application in medical education is concentrated in Europe and the USA, with limited studies in developing countries, likely due to the technological and financial demands of metaverse implementation. This disparity indicates that widespread adoption of metaverse technology in global medical education may face significant challenges. However, technological advancements could help overcome these barriers, improving the feasibility of metaverse utilisation.

This study represents the first systematic evaluation of the metaverse’s role in medical education, offering a novel contribution to the field. To maintain rigorous standards, only randomised controlled trials (RCTs) were included, excluding case–control and cross-sectional studies. The findings provide a theoretical foundation to support and guide future research. Future RCTs with larger sample sizes could yield more generalised and robust insights into the metaverse’s effectiveness in medical education.

## Ethics approval and consent to participate

Not applicable.

## Data availability statement

The data used to support the findings of this study are included within the article.

## Funding

The study did not receive any specific funding from funding agencies in the public, commercial or non-profit sectors.

## CRediT authorship contribution statement

**Qian Li:** Writing – review & editing, Writing – original draft, Software, Methodology, Investigation, Formal analysis, Data curation, Conceptualization. **Hui Duan:** Software, Methodology, Formal analysis, Data curation. **Xinxu Zhou:** Software, Methodology, Formal analysis, Data curation. **Xiaobin Sun:** Writing – review & editing, Supervision, Project administration, Methodology. **Lan Tao:** Writing – review & editing, Supervision, Project administration, Methodology. **Xiuying Lu:** Writing – review & editing, Project administration, Methodology, Conceptualization.

## Declaration of competing interest

The authors declare that they have no known competing financial interests or personal relationships that could have appeared to influence the work reported in this paper.

## Appendix Supplementary materials

Image, application 1

## References

[1] El Naggar M.A., Almaeen A.H. (2020). Student’s perception towards medical-simulation training as a method for clinical teaching [J]. J Pak Med Assoc.

[2] (2020). 3RD T S. The art of teaching, training, and putting the scalpel in residents' Hands [J]. Facial Plast Surg Clin North Am.

[3] Henrique-Sanches B C, Cecilio-Fernandes D., Costa R.R.D.O., Almeida R.G.D.S., Etchegoyen F.F., Mazzo A. (2024). Implications of clinical simulation in motivation for learning: scoping review [J]. Einstein (São Paulo).

[4] Sandrone S. (2022). Medical education in the metaverse [J]. Nat. Med.

[5] Zhang X., Chen Y., Hu L., Wang Y. (2022). The metaverse in education: definition, framework, features, potential applications, challenges, and future research topics [J]. Front Psychol.

[6] Guo H., Gao W. (2022). Metaverse-powered experiential situational English-teaching design: an emotion-based analysis method [J]. Front Psychol.

[7] Siyaev A., Jo G-S (2021). Towards aircraft maintenance metaverse using speech interactions with virtual objects in mixed reality [J]. Sensors.

[8] Wei H., Tang L., Wang W., Zhang J. (2022). Home environment augmented reality system based on 3D reconstruction of a single furniture picture [J]. Sensors.

[9] Lyu Z., Fridenfalk M. (2023). Digital twins for building industrial metaverse [J]. J Adv Res.

[10] Cerci P., Kendirlinan R., Tunakan Dalgiç C. (2023). The perspective of allergy and immunology specialists on the innovations of metaverse: A survey study [J]. Allergol Immunopathol (Madr).

[11] Ford T J, Buchanan D M, Azeez A. (2023). Taking modern psychiatry into the metaverse: integrating augmented, virtual, and mixed reality technologies into psychiatric care [J]. Front Digital Health.

[12] Kim D-J, Jo G., Koo J-H (2022). Development of a simulator capable of generating age-specific pulse pressure waveforms for medical palpation training [J]. Appl Sci.

[13] Almarzouqi A., Aburayya A., Salloum S A (2022). Prediction of user’s intention to use metaverse system in medical education: A hybrid SEM-ML learning approach [J]. IEEE Access.

[14] Kim Y., Kim M.Y. (2023). Effects of metaverse-based career mentoring for nursing students: a mixed methods study [J]. BMC Nurs.

[15] Moro C. (2023). Utilizing the metaverse in anatomy and physiology [J]. Anat Sci Educ.

[16] Greuter L., De Rosa A., Cattin P., Croci D.M., Soleman J., Guzman R. (2021). Randomized study comparing 3D virtual reality and conventional 2D on-screen teaching of cerebrovascular anatomy [J]. Neurosurg Focus.

[17] Bogomolova K., Van Der Ham I J M, Dankbaar M E W (2020). The effect of stereoscopic augmented reality visualization on learning anatomy and the modifying effect of visual-spatial abilities: a double-center randomized controlled trial [J]. Anat Sci Educ.

[18] Stojanovska M., Tingle G., Tan L. (2019). Mixed reality anatomy using Microsoft HoloLens and cadaveric dissection: a comparative effectiveness study [J]. Med Sci Educ.

[19] Liu S., Xie M., Zhang Z. (2021). A 3D hologram with mixed reality techniques to improve understanding of pulmonary lesions caused by COVID-19: randomized controlled trial [J]. J Med Internet Res.

[20] Rai A S, Rai A S, Mavrikakis E. (2017). Teaching binocular indirect ophthalmoscopy to novice residents using an augmented reality simulator [J]. Canad J Ophthalmol.

[21] Logishetty K., Rudran B., Cobb J P (2019). Virtual reality training improves trainee performance in total hip arthroplasty: a randomized controlled trial [J]. Bone Joint J.

[22] Blumstein G., Zukotynski B., Cevallos N. (2020). Randomized trial of a virtual reality tool to teach surgical technique for tibial shaft fracture intramedullary nailing [J]. J Surg Educ.

[23] Crockatt W.K., Confino J.E., Kopydlowski N.J., Jobin C.M., Levine W.N. (2023). Comparing skill acquisition and validity of immersive virtual reality with cadaver laboratory sessions in training for reverse total shoulder arthroplasty [J]. JBJS Open Access.

[24] Lohre R., Bois A.J., Athwal G.S., Goel D.P., Canadian Shoulder and Elbow Society (CSES) (2020). Improved complex skill acquisition by immersive virtual reality training [J]. J Bone and Joint Surg.

[25] Mckinney B., Dbeis A., Lamb A. (2022). Virtual reality training in unicompartmental knee arthroplasty: A randomized, blinded trial [J]. J Surg Educ.

[26] Orland M D, Patetta M J, Wieser M. (2020). Does virtual reality improve procedural completion and accuracy in an intramedullary tibial nail procedure? A randomized control trial [J]. Clin Orthopaed Relat Res.

[27] Birrenbach T., Zbinden J., Papagiannakis G. (2021). Effectiveness and utility of virtual reality simulation as an educational tool for safe performance of COVID-19 diagnostics: prospective, randomized pilot trial [J]. JMIR Serious Games.

[28] Heo S., Moon S., Kim M., Park M., Cha W.C., Son M.H. (2022). An augmented reality–Based guide for mechanical ventilator setup: prospective randomized pilot trial [J]. JMIR Serious Games.

[29] Plotzky C., Loessl B., Kuhnert B. (2023). My hands are running away – learning a complex nursing skill via virtual reality simulation: a randomised mixed methods study [J]. BMC Nurs.

[30] Schoeb D S, Schwarz J., Hein S. (2020). Mixed reality for teaching catheter placement to medical students: a randomized single-blinded, prospective trial [J]. BMC Med Educ.

[31] Cumpston M., Li T., Page M J (2019). Updated guidance for trusted systematic reviews: a new edition of the Cochrane Handbook for Systematic Reviews of Interventions [J]. Cochrane Database Syst Rev.

[32] Tadlock M D, Olson E J, Gasques D. (2022). Mixed reality surgical mentoring of combat casualty care related procedures in a perfused cadaver model: initial results of a randomized feasibility study [J]. Surgery.

[33] Wang C., Daniel B.K., Asil M., Khwaounjoo P., Cakmak Y.O. (2020). A randomised control trial and comparative analysis of multi-dimensional learning tools in anatomy [J]. Sci Rep.

[34] Nagayo Y., Saito T., Oyama H. (2022). Augmented reality self-training system for suturing in open surgery: a randomized controlled trial [J]. Int J Surg.

[35] Pulijala Y., Ma M., Pears M. (2018). Effectiveness of immersive virtual reality in surgical training: a randomized control trial [J]. J Oral Maxillofac Surg.

[36] Veer V., Phelps C., Moro C. (2022). Incorporating mixed reality for knowledge retention in physiology, anatomy, pathology, and pharmacology interdisciplinary education: a randomized controlled trial [J]. Med Sci Educ.

[37] Alam I., Garg K., Kumar A G (2024). Beyond traditional training: exploring the benefits of virtual reality simulator in lumbar pedicle screw insertion: a randomized controlled trial [J]. World Neurosurg.

[38] Çetinkaya Uslusoy E, Aydinli A., Durna F. (2024). Enhancing learning motivation and academic achievement in nursing students through metaverse-based learning: a randomized controlled study [J]. Jpn J Nurs Sci.

[39] Brix L D, Skjodt-Jensen A M, Jensen T H (2025). Enhancing nursing students' self-reported self-efficacy and professional competence in basic life support: the role of virtual simulation prior to high-fidelity training [J]. Teach Learning Nurs.

[40] Dubinski D., Won S Y, Hardung C. (2025). Enhancing surgical education for medical students through virtual reality: the digital Surgical Operating Theatre tour [J]. World Neurosurg.

[41] D'aiello A F, Cabitza F., Natali C. (2023). The effect of holographic heart models and mixed reality for anatomy learning in congenital heart disease: an exploratory study [J]. J Med Syst.

[42] Liaw S.Y., Rusli K.D.B., Ooi S.W., Lau T.C., Tam W.W.S., Chua W.L. (2020). Nurse-physician communication team training in virtual reality versus live simulations: randomized controlled trial on team communication and teamwork attitudes [J]. J Med Internet Res.

[43] Yang S-Y, Kang M-K (2022). Efficacy testing of a Multi-access metaverse-based early onset schizophrenia nursing simulation program: a quasi-experimental study [J]. Int J Environ Res Public Health.

[44] Lamb A., Mckinney B., Frousiakis P. (2023). A comparative study of traditional technique guide versus virtual reality in orthopedic trauma training [J]. Adv Med Educ Pract.

[45] Guha P., Lawson J., Minty I., Kinross J., Martin G. (2023). Can mixed reality technologies teach surgical skills better than traditional methods? A prospective randomised feasibility study [J]. BMC Med Educ.

[46] Francis E R, Bernard S., Nowak M L (2020). Operating room virtual reality immersion improves self-efficacy amongst preclinical physician assistant students [J]. J Surg Educ.

[47] Morimoto T., Kobayashi T., Hirata H. (2022). XR (Extended Reality: Virtual Reality, Augmented Reality, Mixed Reality) Technology in Spine Medicine: Status quo and Quo vadis [J]. J Clin Med.

[48] Shaffer K. (2019). Why we need a guide to new teaching methods now" [J]. Acad Radiol.

[49] Guetterman T C, Sakakibara R., Baireddy S. (2019). Medical students' Experiences and outcomes using a virtual Human simulation to improve communication skills: mixed methods study [J]. J Med Internet Res.

[50] Moro C., Štromberga Z., Raikos A. (2017). The effectiveness of virtual and augmented reality in health sciences and medical anatomy [J]. Anat Sci Educ.

[51] Ebina K., Abe T., Higuchi M. (2021). Motion analysis for better understanding of psychomotor skills in laparoscopy: objective assessment-based simulation training using animal organs [J]. Surg Endosc.

[52] Lorenzo-Alvarez R., Rudolphi-Solero T., Ruiz-Gomez M J (2019). Game-based learning in Virtual worlds: A multiuser online Game for medical undergraduate radiology education within second life [J]. Anat Sci Educ.

[53] Wu Q., Wang Y., Lu L. (2022). Virtual simulation in undergraduate medical education: A scoping review of recent practice [J]. Front Med (Lausanne).

[54] Mcwilliam A., Scarfe P. (2023). The metaverse and oncology [J]. Clin Oncol (R Coll Radiol).

[55] Houze-Cerfon C H, Vaissié C., Gout L. (2019). Development and evaluation of a virtual research environment to improve quality of care in overcrowded emergency departments: observational study [J]. JMIR Serious Games.

[56] Aardoom J J, Hilt A D, Woudenberg T. (2022). A preoperative virtual reality app for patients scheduled for cardiac catheterization: pre-post questionnaire study examining feasibility, usability, and acceptability [J]. JMIR Cardio.

[57] Benrimoh D., Chheda F D, Margolese HC. (2022). The best predictor of the future-the metaverse, mental health, and lessons learned from current technologies [J]. JMIR Ment Health.

