# Supplementary material for: The use of metaverse in medical education: A systematic review
Source: Clin Med (Lond). 2025 Apr 15;25(3):100315. doi: 10.1016/j.clinme.2025.100315 (PMC12059326; doi:10.1016/j.clinme.2025.100315)
Supplement: Supplementary file 1 [file mmc1.docx]

TableS1 search strategy

PubMed-647（Retrieved March 9, 2025）

(((Metaverse) OR ((("Augmented Reality"[Mesh]) OR ((((((((Augmented Realities) OR (Realities, Augmented)) OR (Reality, Augmented)) OR (Mixed Reality)) OR (Mixed Realities)) OR (Realities, Mixed)) OR (Reality, Mixed)) OR (artificial reality))) AND (((((((((((((Virtual Reality[MeSH Terms]) OR (Reality, Virtual)) OR (Virtual Reality, Educational)) OR (Educational Virtual Realities)) OR (Educational Virtual Reality)) OR (Reality, Educational Virtual)) OR (Virtual Realities, Educational)) OR (Virtual Reality, Instructional)) OR (Instructional Virtual Realities)) OR (Instructional Virtual Reality)) OR (Realities, Instructional Virtual)) OR (Reality, Instructional Virtual)) OR (Virtual Realities, Instructional)))) AND (((((Education, Medical[MeSH Terms]) OR (Medical Education)) OR (Teaching)) OR (Training)) OR (Learning))) AND ((((((cross-sectional) OR (cohort)) OR (randomized controlled trial)) OR (RCT)) OR (trial)) OR (random*))

Embase-218（Retrieved March 9, 2025）

((Metaverse or ((Augmented Reality or (Augmented Realities or Realities, Augmented or Reality, Augmented or Mixed Reality or Mixed Realities or Realities, Mixed or Reality, Mixed or artificial reality)) and (Virtual Reality or Reality, Virtual or Virtual Reality, Educational or Educational Virtual Realities or Educational Virtual Reality or Reality, Educational Virtual or Virtual Realities, Educational or Virtual Reality, Instructional or Instructional Virtual Realities or Instructional Virtual Reality or Realities, Instructional Virtual or Reality, Instructional Virtual or Virtual Realities, Instructional))) and (Education, Medical or Medical Education or Teaching or Training or Learning) and (cross-sectional or cohort or randomized controlled trial or RCT or trial or random*)).af.

Cochrane-90（Retrieved March 9, 2025）

((Metaverse or ((Augmented Reality or (Augmented Realities or Realities, Augmented or Reality, Augmented or Mixed Reality or Mixed Realities or Realities, Mixed or Reality, Mixed or artificial reality)) and (Virtual Reality or Reality, Virtual or Virtual Reality, Educational or Educational Virtual Realities or Educational Virtual Reality or Reality, Educational Virtual or Virtual Realities, Educational or Virtual Reality, Instructional or Instructional Virtual Realities or Instructional Virtual Reality or Realities, Instructional Virtual or Reality, Instructional Virtual or Virtual Realities, Instructional))) and (Education, Medical or Medical Education or Teaching or Training or Learning) and (cross-sectional or cohort or randomized controlled trial or RCT or trial or random*)).af.

Web Of Science(Preprint Citation Index)-30（Retrieved March 9, 2025）

(((Metaverse) OR (((Augmented Reality) OR ((((((((Augmented Realities) OR (Realities, Augmented)) OR (Reality, Augmented)) OR (Mixed Reality)) OR (Mixed Realities)) OR (Realities, Mixed)) OR (Reality, Mixed)) OR (artificial reality))) AND (((((((((((((Virtual Reality) OR (Reality, Virtual)) OR (Virtual Reality, Educational)) OR (Educational Virtual Realities)) OR (Educational Virtual Reality)) OR (Reality, Educational Virtual)) OR (Virtual Realities, Educational)) OR (Virtual Reality, Instructional)) OR (Instructional Virtual Realities)) OR (Instructional Virtual Reality)) OR (Realities, Instructional Virtual)) OR (Reality, Instructional Virtual)) OR (Virtual Realities, Instructional)))) AND (((((Education, Medical) OR (Medical Education)) OR (Teaching)) OR (Training)) OR (Learning))) AND ((((((cross-sectional) OR (cohort)) OR (randomized controlled trial)) OR (RCT)) OR (trial)) OR (random*)) (Topic)

Web Of Science(core collection)-1882

(((Metaverse) OR (((Augmented Reality) OR ((((((((Augmented Realities) OR (Realities, Augmented)) OR (Reality, Augmented)) OR (Mixed Reality)) OR (Mixed Realities)) OR (Realities, Mixed)) OR (Reality, Mixed)) OR (artificial reality))) AND (((((((((((((Virtual Reality) OR (Reality, Virtual)) OR (Virtual Reality, Educational)) OR (Educational Virtual Realities)) OR (Educational Virtual Reality)) OR (Reality, Educational Virtual)) OR (Virtual Realities, Educational)) OR (Virtual Reality, Instructional)) OR (Instructional Virtual Realities)) OR (Instructional Virtual Reality)) OR (Realities, Instructional Virtual)) OR (Reality, Instructional Virtual)) OR (Virtual Realities, Instructional)))) AND (((((Education, Medical) OR (Medical Education)) OR (Teaching)) OR (Training)) OR (Learning))) AND ((((((cross-sectional) OR (cohort)) OR (randomized controlled trial)) OR (RCT)) OR (trial)) OR (random*)) (Topic)
